# Ready, Willing and Able? An Investigation of the Theory of Planned Behaviour in Help-Seeking for a Community Sample with Current Untreated Depressive Symptoms

**DOI:** 10.1007/s11121-020-01099-2

**Published:** 2020-03-05

**Authors:** S. Tomczyk, G. Schomerus, S. Stolzenburg, H. Muehlan, S. Schmidt

**Affiliations:** 1 Institute of Psychology, Department Health and Prevention, University of Greifswald, Robert-Blum-Str. 13, 17487 Greifswald, Germany; 2 Medical Faculty, Department of Psychiatry, University of Leipzig, Leipzig, Germany; 3 Clinic for Psychosomatic Medicine and Psychotherapy, Helios Hanseklinikum Stralsund, Stralsund, Germany

**Keywords:** Help-seeking, Theory of planned behaviour, Depression, Mental health, General population

## Abstract

Applying health behaviour change models, such as the theory of planned behaviour (TPB), to help-seeking for mental health problems can address the deficit in health care utilisation. However, previous studies largely focused on help-seeking intentions and not behaviour, which might be problematic due to the intention-behaviour gap. Hence, TPB and help-seeking were examined in a German community sample with current untreated depressive symptoms: 188 adults (*M*_age_ = 50.34; SD = 16.19; 70.7% female) participated in a baseline interview and survey measuring components of the TPB (attitudes, subjective norms and perceived behavioural control) and help-seeking intentions. They reported actual help-seeking from mental health professionals via telephone surveys 3 and 6 months later. To better understand the potential gap between help-seeking intentions and behaviour and to investigate the contributions of readiness, willingness and ability to seek help, two path models were constructed in accordance with the TPB controlling for covariates. Attitudes (*β* = .24), subjective norms (*β* = .25) and self-efficacy (*β* = .15) were significantly associated with intentions (*R*^2^ = 26%), which predicted help-seeking (Cox and Snell’s pseudo-*R*^2^ = 23%); controllability did not predict help-seeking. In sum, the TPB provides a reliable framework to explore help-seeking behaviour for mental health problems. Based on these findings, prevention efforts should focus on readiness and willingness to seek help (e.g. foster positive attitudes and social support of treatment). However, the role of ability, operationalised as perceived behavioural control and (perceived) barriers to help-seeking, warrants further research, as self-efficacy but not controllability was associated with help-seeking.

**Electronic supplementary material:**

The online version of this article (10.1007/s11121-020-01099-2) contains supplementary material, which is available to authorized users.

## Introduction

According to the World Health Organization, depression is one of the main health-related burdens of society. It currently represents a major cause of non-fatal health loss worldwide (World Health Organization 2017). While seeking adequate treatment proves to be highly effective in battling depression (Cuijpers et al. 2013), a minority of people with depression actually seeks professional help. Therefore, numerous studies have investigated attitudinal and structural barriers to help-seeking (e.g. Clement et al. 2015; Gonzalez et al. 2005; Schnyder et al. 2017; Schomerus et al. 2009b). Attitudinal barriers refer to cognitive, emotional and motivational aspects, such as negative attitudes towards health services or mental illness stigma (Clement et al. 2015; Schnyder et al. 2017), whereas structural barriers comprise aspects such as low-socioeconomic status, financial costs of health services, cultural background (e.g. informal help-seeking is preferred over professional help in several Eastern cultures) and availability of mental health services (e.g. low density of services in rural areas) (Altweck et al. 2015; Mak and Davis 2014; Mo and Mak 2009; Sareen et al. 2007). To tackle the deficit in treatment utilisation, it is important to closely inspect the help-seeking process and to identify starting points for prevention research and practice.

### Help-Seeking for Depressive Symptoms

Help-seeking can be defined as a psychosocial form of health behaviour (Nudelman and Shiloh 2015), where one discloses a problem to get external help. In contrast to health behaviours like physical activity, help-seeking depends on an external source (Cornally and McCarthy 2011). In mental health care, seeking external help is one of the first conscious steps towards treatment. Following the Goldberg-Huxley model (Huxley 1996), presenting one’s symptoms to a medical professional is crucial to the treatment process and the referral to secondary care. Given that depressive syndromes are among the most prevalent mental disorders in the general population (Alonso et al. 2004; Bretschneider et al. 2017), investigating the help-seeking process for depressive symptoms is a public health concern.

However, previous studies on help-seeking in this area predominantly investigated behavioural intentions as a proxy for actual help-seeking (Clement et al. 2015; Schnyder et al. 2017). This is problematic, as behavioural research points to a gap between intention and behaviour (Sheeran and Webb 2016) for instance, due to individual forgetfulness or a lack of opportunity to perform (Lang 2005). From a psychological perspective, help-seeking behaviour can be characterised by multiple aspects (Miller and Rollnick 2002; Rickwood and Thomas 2012; Romano and Netland 2008; White et al. 2018), that is (1) assessing a psychological or emotional problem and recognizing the need for external help (i.e. recognition), (2) identifying appropriate sources of help and being open to seeking them out (i.e. readiness), (3) feeling able to seek external help despite potential barriers (i.e. ability), (4) making plans and forming intentions to seek help by prioritizing help-seeking over alternative behaviours (i.e. willingness) and (5) contacting external sources and thereby seeking help (i.e. behaviour). To successfully arrive at behavioural performance, recognition, readiness, ability and willingness have to align towards help-seeking. Therefore, a misalignment of these aspects is a potential cause of the observed intention-behaviour gap.

Several psychological models of health behaviour change have applied this perspective to help-seeking, that is the change from not seeking help to seeking help. However, a recent literature review (White et al. 2018) states that the theory of planned behaviour is ‘the only model that potentially addresses all aspects’ (p. 68), while other models, such as the transtheoretical model of health behaviour change (Prochaska and DiClemente 1992; Prochaska and Velicer 1997), do not (an extended discussion of advantages and disadvantages of different health behaviour models in this context is beyond the scope of this article). Thus, the theory of planned behaviour has unique potential to bridge the gap between intention and behaviour regarding help-seeking for mental health problems.

### Using the Theory of Planned Behaviour to Explore Help-Seeking for Depressive Symptoms and the Intention-Behaviour Gap

The theory of planned behaviour (TPB) describes the associations between cognitive factors, behavioural intentions and their implementation (Ajzen 1991, 2002). Among its core components are subjective norms, attitudes towards health behaviour and perceived behavioural control. These components are supposed to predict intention, which leads to behavioural performance. Large meta-analyses of the TPB (Armitage and Conner 2001; McEachan et al. 2011) empirically support the model and its components across a variety of health behaviours.

The components of the TPB can be linked to the stages of the help-seeking process and thus address the intention-behaviour gap, in the following ways: Subjective norms include beliefs about what other people think about a behaviour, for example how strongly other people practice (descriptive norms) and encourage (injunctive norms) help-seeking. This aspect is connected to recognition and readiness, as it represents awareness of social support towards professional help-seeking for current mental health problems. Attitudes focus on the evaluation of a behaviour, for example whether seeking help for depressive symptoms is perceived as being harmful or beneficial. This aspect is connected to readiness, as it reflects individual values and perceived benefits of behavioural change towards help-seeking. However, it also has an impact on willingness, as it reflects the instrumentality of seeking help for attaining one’s goals and values, which informs the preference of help-seeking over alternative behaviours according to motivational enhancement theory (Miller and Rollnick 2002). Perceived behavioural control consists of self-efficacy (the confidence to be able to perform a behaviour) and controllability (the extent of personal control over behavioural performance) and thus represents the aspect of ability. Although self-efficacy and controllability are intertwined, their theoretical and empirical differentiation is important (Ajzen 2002; Kleinberg et al. 2013): For example, if mental health services are not easily available, for instance, in rural areas, perceived controllability might be low, while self-efficacy might not be affected (Bandura 2004). These three core components of the TPB inform behavioural intention, which in turn predicts performance (Romano and Netland 2008). In line with the psychological aspects of the help-seeking process, intention is a representation of willingness, in that it is a conscious plan to communicate one’s mental health problems to external sources with help-seeking having instrumental congruency with one’s values. Finally, perceived behavioural control is also hypothesised to directly influence behaviour, that is self-efficacy captures one’s confidence to perform in spite of challenging circumstances, such as scarce financial resources, while controllability captures the perceived opportunities to do so (Ajzen 2002).

#### Empirical Support for the TPB in Help-Seeking

To date, several studies have investigated components of the TPB in help-seeking for depressive symptoms (Bohon et al. 2016; Hui et al. 2015; Mak and Davis 2014; Mo and Mak 2009; Schomerus et al. 2009a). Overall, these studies report positive associations between attitudes, subjective norm, perceived behavioural control and help-seeking. Attitudes were the strongest predictor of intention in all but one study, where perceived behavioural control showed the strongest associations with intentions (Mak and Davis 2014).

However, a closer look at these studies reveals several difficulties and open questions. The study by Hui et al. (2015) did not focus on help-seeking per se but an online campaign promoting positive help-seeking attitudes. The remaining studies focused on help-seeking intentions, but they did not capture actual help-seeking behaviour; therefore, the question of the intention-behaviour gap remains unanswered (Sheeran and Webb 2016). Moreover, they mostly investigated healthy samples and used vignettes of mental illnesses to elicit attitudes towards help-seeking, despite their methodological limitations, as vignettes can lead to biased estimates, for example by evoking gender stereotypes (Swami 2012). Furthermore, these studies investigated diverse groups: among college students (Bohon et al. 2016), subjective norms were strongly associated with attitudes and perceived behavioural control, but not help-seeking intentions. In a subgroup of the German population with depressive symptoms, on the other hand, Schomerus et al. (2009a) found that perceived behavioural control did not predict intentions. However, they did not discern its subcomponents self-efficacy and controllability to further explore this observation. Hence, the impact of either one on help-seeking remains unclear, despite a differential impact being suggested by Ajzen himself (2002).

## Study Strengths and Research Objectives

To add to the literature, this study aims to prospectively investigate help-seeking in a German community sample with currently untreated depressive symptoms. In Germany, evidence-based mental health treatment is covered by statutory insurance, thus financial barriers to treatment are less significant, for instance, compared with the USA (Kamke 1998; Sareen et al. 2007). Moreover, ethnical and cultural differences regarding help-seeking are less pronounced, as epidemiological studies point to similar rates of mental illness and health care seeking among migrants and autochthonous populations (Bretschneider et al. 2017). Despite these differences, however, the study holds promise for the following three areas:

1. Addressing the intention-behaviour gap by focussing on actual help-seeking behaviour instead of help-seeking intentions or recommendations.
2. Investigating help-seeking via a longitudinal community sample with untreated depressive symptoms, which provides higher ecological validity than previous vignette-based studies.
3. Examining the TPB, including the differentiation of perceived behavioural control (self-efficacy and controllability), in predicting help-seeking intentions and behaviour.

## Methods

### Sample

To recruit people from the German population with currently untreated depressive symptoms, advertisements were placed online and offline, for instance, in local newspapers. Advertisements described depressive symptoms without using diagnostic labels or psychiatric terminology (see the Appendix of Stolzenburg et al. 2017 for the advertisement text) to avoid stigmatizing processes associated with terms of mental illness. Overall, 429 participants contacted the study centre and completed an initial telephone screening for eligibility. Eligibility criteria included being at least 18 years of age, not receiving psychiatric or psychotherapeutic treatment within the past 6 months and having a score of at least 8 on the German version of the Patient Health Questionnaire-Depression (PHQ-D; Gräfe et al. 2004). This cutoff was deliberately set below the threshold for moderate depression (10 points), because participants may have been reluctant to disclose the full extent of their symptoms in a telephone interview. Nevertheless, a meta-analysis (Manea et al. 2012) reported sufficient sensitivity (.82) and specificity (.83) for diagnosing major depressive disorder based on a cutoff of ≥ 8 points using the PHQ-D.

Eligible participants (*N* = 266) were then invited to complete a survey and to undergo the Mini-International Neuropsychiatric Interview (M.I.N.I.; Sheehan et al. 1998), conducted by three clinically trained psychologists. Participants received 30 € for their participation.

Of this eligible sample, 233 participants partook in the interview and the survey. Thereof, four participants were excluded, because they received professional mental health treatment at the time of the assessment, and 22 participants, because they did not meet the diagnostic criteria for any mental disorder according to the interview (i.e. the M.I.N.I.).

As a result, the baseline sample comprised 207 participants. Finally, two follow-up surveys were conducted via telephone (3 and 6 months after the baseline assessment) focussing on participants’ help-seeking behaviour regarding their mental health problems. In sum, 188 participants (*M*_age_ = 50.34; SD = 16.19; 70.7% female) completed one or both of the follow-ups. Regarding their diagnosis, recurrent depressive disorder (*n* = 64), depressive episode (*n* = 56) and other persistent mood disorder (*n* = 33) were the most prevalent mental disorders. Data collection lasted from November 2015 to July 2016. All persons gave informed consent prior to study inclusion. This study was approved by the Ethics Committee of the University Medicine Greifswald and was therefore conducted in accordance with the Declaration of Helsinki.

### Measures

#### Attitudes Towards Help-Seeking, Subjective Norms and Perceived Behavioural Control

Attitudes towards help-seeking, subjective norms and perceived behavioural control were based on a TPB questionnaire developed by Schomerus et al. (2009a) that was validated regarding psychometric properties and feasibility in a representative survey of the German population. In accordance with Ajzen’s recommendations (Ajzen 1991, 2002), the items used a consistent wording of help-seeking for all aspects of the TPB and provided a clear point of reference for the behaviour in question. Therefore, the items were rephrased in the current study to refer to respondents’ self-reported mental health problems (see Table S1 for the TPB questionnaire, available online). All items measuring subjective norms and perceived behavioural control were rated on a seven-point scale from 1 (*strongly disagree*) to 7 (*strongly agree*). Sum scores were calculated for each scale with higher values representing a more positive attitude, affirmative norms and higher perceived behavioural control.

To measure attitudes (*α* = .69), participants indicated their attitude towards seeking professional help on three pairs of bipolar items (e.g. *good*—*bad*) on a seven-point scale. Subjective norms (*α* = .84) were measured with four items, perceived behavioural control (*α* = .49) was also measured with four items. After excluding one item from this scale (I need external support to receive professional help for my problems), internal consistency was acceptable (*α* = .74). In the original study, Schomerus et al. (2009a) also observed low internal consistency of the perceived behavioural control scale (*α* = .54); however, they did not remove any item from the scale. Nevertheless, a confirmatory factor analysis (see Statistical Analysis for details on model fit criteria) of the original TPB model (including said item) revealed insufficient model fit in the current study (*χ*^2^ (36) = 98.59, *p* < .001; CFI = .90; TLI = .84; RMSEA = .09 [.07, .11]). It can be assumed that the excluded item does not fit the scale, because it refers to the passive aspect of receiving help rather than the active aspect of seeking help, which was the focus of this study. Conceptually, receiving help embodies the desired consequence of seeking it (Cornally and McCarthy 2011). For these reasons, the item was excluded from the analysis.

To further explore potential differences between self-efficacy and controllability and their association with help-seeking, two separate path models were tested (model 1 and model 2). Measures of perceived behavioural control were included as either an overall score (model 1; all three remaining items) or two separate scores (model 2) for self-efficacy (one item) and controllability (two items). To test the feasibility of this approach, confirmatory factor analyses of TPB components for model 1 (*χ*^2^ (32) = 63.46, *p* < .01; CFI = .95; TLI = .91; RMSEA = .07 [.05, .10]) and model 2 (*χ*^2^ (30) = 55.98, p < .01; CFI = .96; TLI = .92; RMSEA = .07 [.04, .09]) showed acceptable model fit for both models.

#### Help-Seeking Intentions and Behaviour

To assess intentions, participants were asked how likely they would seek help for their problems in the next 3 months from fifteen different sources, for instance, friends, a psychologist or a priest, on a scale from 1 (*extremely unlikely*) to 7 (*extremely likely*). This response format has previously been validated for general help-seeking (Wilson et al. 2005). The current study focused on help-seeking from mental health professionals, that is seeing a psychiatrist, psychotherapist or clinical psychologist. All three items were strongly correlated (*r* = .68 to *r* = .78) and showed high internal consistency (*α* = .89). In accordance with previous research (Stolzenburg et al. 2018), these items were combined into a single variable with the highest value of any of the three items representing the strongest intention to seek professional help. To account for an average latency of 12.5 weeks between initial contact and first appointment with a mental health professional in Germany (Bundespsycho-therapeutenkammer (BPtK) 2011), baseline intentions were chosen as a proxy for help-seeking intentions regarding the entire follow-up period of 6 months.

For help-seeking behaviour, participants were asked whether they had sought help in the past 3 months from several different sources, 3 and 6 months after the baseline assessment. Items were scored as 1 (*yes*) or 0 (*no*). Consistent with help-seeking intentions, ratings of help-seeking regarding mental health professionals (psychiatrist, psychotherapist, clinical psychologist) were combined into a single dichotomous variable indicating whether participants had sought any professional help (vs. none). To account for the latency between initial contact and actual appointment, responses were combined across both follow-ups to obtain a single indicator of help-seeking in the 6 months following the baseline assessment.

#### Covariates

To test the stability of the TPB path models, covariates were included that previous research identified as confounders of the help-seeking process (e.g. Clement et al. 2015; Sareen et al. 2007; Schnyder et al. 2017; Schomerus et al. 2009a). Age and gender (0 (*male*), 1 (*female*)) were measured via single variables. To approximate socioeconomic status, monthly income was measured on a scale from 1 (*0–500€*) to 6 (*2501€ or more*) and level of education, that is successfully completed years of secondary education were also assessed. In Germany, the highest level of secondary education is required to enter a university. Therefore, two dummy variables were created to reflect average (*10 to 11 school years*) and highest secondary education (*12 to 13 school years*), with lowest secondary education (*< 10 school years*) as the remaining reference group. Additionally, participants stated whether they had ever been in professional treatment for any mental health problems before (1 (*yes*), 0 (*no*)).

Finally, because depressive symptoms were used as an inclusion criterion and thus highly prevalent in the analysis sample, depression severity was included as a covariate. It was measured via the sum score of the depression scale (*α* = .79) of the PHQ-D (Gräfe et al. 2004). The questionnaire assesses depressive symptoms via nine items corresponding to the DSM-IV criteria on a four-point scale from 0 (*not at all*) to 3 (*nearly every day*). A higher score indicates greater depression severity.

### Statistical Analysis

First, baseline data between was compared between participants with (*N* = 188) and without complete outcome data (*n* = 19) and the remaining analysis sample was checked for missing data. Second, a descriptive analysis of baseline data was conducted. Third, bivariate pairwise point-biserial and Pearson’s correlations were used to test assumptions about the associations between variables. Fourth, two path models were constructed in accordance with the theory of planned behaviour. Model 1 included five components (subjective norms, attitudes towards help-seeking, perceived behavioural control, help-seeking intention and help-seeking). Model 2 considered the internal differentiation of perceived behavioural control (self-efficacy and controllability as separate components) and thus included six components. Both models were tested with and without controlling for covariates (age, gender, monthly income, level of education, previous mental health treatment and severity of depressive symptoms) by including covariates as predictors of intention and behaviour and modelling covariance between covariates and the remaining TPB components. Model fit was evaluated via the overall goodness-of-fit chi-squared test (a non-significant result indicates good overall fit to the data), the comparative fit index and the Tucker-Lewis index (CFI and TLI; a value near 1 indicates good fit) and the root mean square error of approximation (RMSEA; a value < 0.05 indicates excellent, < 0.08 acceptable model fit) (Schreiber et al. 2006). Moreover, standardised estimates of path coefficients and the explained variance (*R*^2^) in help-seeking intention were reported. Since behaviour was assessed as a binary outcome variable (none vs. any help), pseudo-*R*^2^ was reported (Cox and Snell 1989). All analyses were based on *α* = .05 and were conducted with SPSS 25 and AMOS 25 (Arbuckle 2017) using complete case analysis for descriptive statistics and full information maximum likelihood estimation for path models.

## Results

Participants that did not complete the follow-up telephone surveys (*n* = 19) were significantly younger (*t*(205) = 2.00, *p <* .05) than remaining participants. In the remaining analysis sample (*N* = 188), there were 3.8% missing values, which an initial test (missing value analysis in SPSS 25) identified as missing completely at random (χ^2^(442) = 454.78, *p* = .327). Overall, 25% of the sample (*n* = 47) sought help from any mental health professional during the 6 months following the baseline assessment. See Table 1 for baseline data of the analysis sample.

**Table 1:** Baseline values of TPB, help-seeking intentions and covariates in a German community sample of adults with currently untreated depressive symptoms (*N* = 188), valid data only. The table lists data for the entire sample (total) and provides values for subsamples of people that did (*n* = 47; ‘yes’) or did not (*n* = 141; ‘no’) seek professional help over the course of 6 months following the baseline assessment

	Help-seeking at any follow-up		
	Yes (% or *M* (SD))	No (% or *M* (SD))	Total (% or *M* (SD))
Gender			
Male	13 (27.7%)	42 (29.8%)	55 (29.3%)
Female	34 (72.3%)	99 (70.2%)	133 (70.7%)
Age (range 18–80)	52.79 (15.80)	49.52 (16.29)	50.34 (16.19)
Monthly income			
0–500 €	5 (10.9%)	23 (16.3%)	28 (15.0%)
501–1000 €	22 (47.8%)	43 (30.5%)	65 (34.8%)
1001–1500 €	12 (26.1%)	40 (28.4%)	52 (27.8%)
1501–2000€	2 (4.4%)	22 (15.6%)	24 (12.8%)
2001–2500 €	4 (8.7%)	8 (5.7%)	12 (6.4%)
2501 € and more	1 (2.2%)	5 (3.6%)	6 (3.2%)
Level of education (school years)			
Lower secondary (9 years)	4 (8.9%)	9 (6.5%)	13 (7.1%)
Average secondary (10 years)	22 (48.9%)	83 (60.1%)	105 (57.4%)
Higher secondary (12 or 13 years)	19 (42.2%)	46 (33.3%)	65 (34.6%)
Previous mental health treatment**			
None	14 (29.8%)	71 (52.2%)	85 (46.4%)
Any	33 (70.2%)	65 (47.8%)	98 (53.6%)
Depression severity** (range 3–27)	14.72 (4.58)	12.14 (4.67)	12.75 (4.75)
TPB^b^ attitudes towards treatment* (range 3–21)	17.45 (4.68)	15.60 (4.21)	16.12 (4.41)
TPB subjective norms*** (range 4–28)	14.64 (7.61)	10.36 (6.15)	11.45 (6.79)
TPB perceived behavioural control (range 3–21)	18.32 (3.59)	17.36 (4.12)	17.60 (4.00)
TPB perceived behavioural control (self-efficacy; range 1–7)**	6.02 (1.48)	5.28 (1.96)	5.47 (1.87)
TPB perceived behavioural control (controllability; range 2–14)	12.29 (2.50)	12.08 (2.88)	12.13 (2.79)
Intention to seek help*** (range 1–7)	4.43 (2.29)	2.54 (1.75)	3.01 (2.07)

*M*, Mean; *SD*, standard deviation

^a^Differences between subsamples were tested via Fisher’s tests for categorical variables, and Student *t* tests for continuous variables

^b^*TPB*, theory of planned behaviour

*p < .05

**p < .01

***p < .001

In the help-seeking group, over 70% had previously been in treatment compared with less than 50% in the non-seeking group. In addition, participants that sought help reported more severe symptoms, and higher intentions to seek help. Furthermore, they had more positive attitudes and subjective norms regarding external help. In terms of perceived behavioural control, there was no overall difference. However, eventual help-seekers reported a higher help-seeking self-efficacy at baseline.

Before building path models, the correlations between the core components of the TPB, help-seeking intentions and behaviour were examined (see Table 2). Help-seeking intentions were positively associated with behaviour. TPB components attitudes, subjective norms and perceived behavioural control were positively associated with intentions. In addition, attitudes, subjective norms and perceived self-efficacy were positively associated with help-seeking behaviour, while the associations were not significant for perceived controllability or the overall score of perceived behavioural control. Self-efficacy and controllability were moderately correlated (*r* = .46, *p* < .01).

**Table 2:** Pairwise point-biserial and Pearson’s correlations between components of the theory of planned behaviour (TPB), help-seeking intentions and help-seeking behaviour regarding professional help (psychologist, psychotherapist or psychiatrist) in a community sample of German adults with currently untreated depressive symptoms (*N* = 188)

	1	2	3	4	5	6	7
1. TPB^a^ attitudes towards treatment	1						
2. TPB subjective norms	.32***	1					
3. TPB perceived behavioural control (overall)	.19*	.15*	1				
4. TPB perceived behavioural control (self-efficacy)	.17*	.20*	.79***	1			
5. TPB perceived behavioural control (controllability)	.15	.07	.91***	.46**	1		
6. Intention to seek help	.37***	.39***	.19*	.25**	.10	1	
7. Help-seeking	.19*	.28***	.10	.17*	.03	.40***	1

^a^*TPB*, theory of planned behaviour

*p < .05

**p < .01

***p < .001

### Path Models of the Theory of Planned Behaviour

**Fig. 1:**
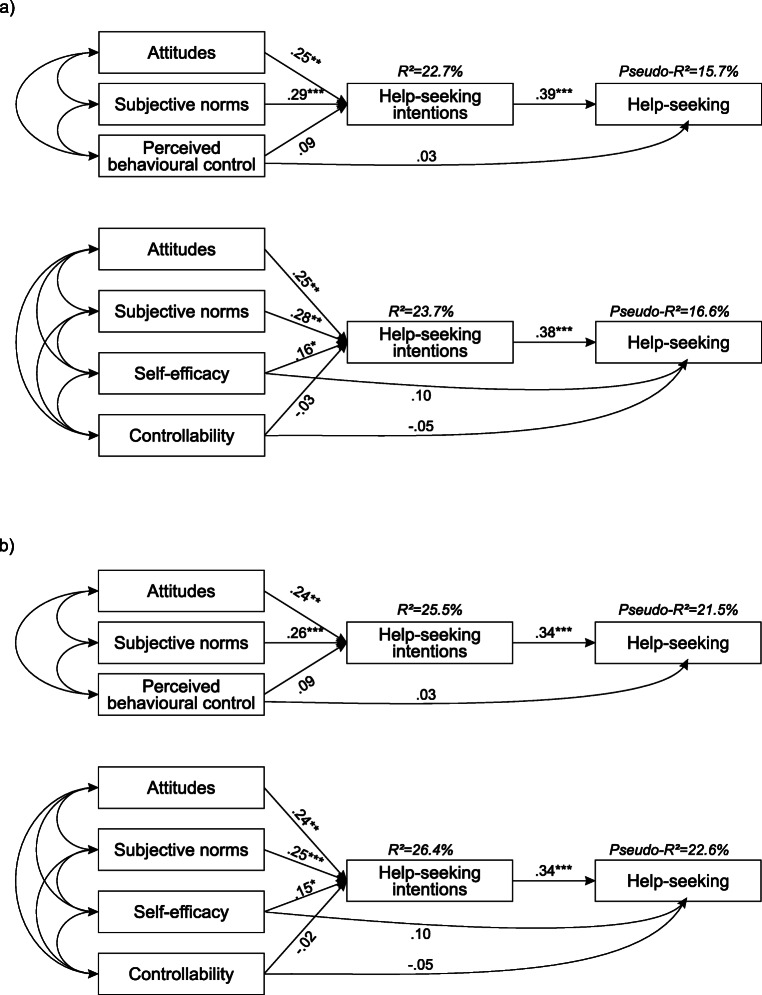
Path models (including standardised path coefficients) of the theory of planned behaviour for professional help-seeking in a community sample of German adults with currently untreated depressive symptoms (*N* = 188), with perceived behavioural control as a single exogenous variable, and represented by its two components self-efficacy and controllability. The path models are **a** not adjusted and **b** adjusted for gender, age, monthly income, level of education, past treatment and severity of depressive symptoms; *p < .05, **p < .01, ***p < .001

As previously discussed, model 1 contains five components (attitudes, subjective norms, perceived behavioural control, help-seeking intentions and behaviour) while model 2 divided perceived behavioural control into perceived self-efficacy and perceived controllability. When controlling for covariates, model 1 showed good model fit (*χ*^2^ (2) = 2.31, *p* = .32; CFI = .99; TLI = .97; RMSEA = .03 [.00, .15]) and explained 25.5% of variation in intention, pseudo-*R*^2^ for help-seeking behaviour was .215. Model 2 also showed good model fit (*χ*^2^ (2) = 2.10, *p* = .35; CFI = 1.00; TLI = .99; RMSEA = .02 [.00, .15]) and explained 26.4% of variation in intention, pseudo-*R*^2^ for help-seeking behaviour was .226. To evaluate the impact of each component of the TPB, standardised path coefficients for path models without (1) and with (2) covariates are printed in Fig. 1 (for path coefficients of covariates, see Table S2, available online).

In both models, attitudes and subjective norms were associated with help-seeking intentions, which predicted help-seeking behaviour. Perceived behavioural control was not associated with help-seeking behaviour in model 1. However, model 2 pointed to an internal differentiation of perceived behavioural control: Higher self-efficacy predicted help-seeking intentions (*β* = .15; *p* < .05) and was by trend positively associated with behaviour, whereas controllability showed weaker, and even negative associations with help-seeking intentions and behaviour.

## Discussion

This is one of the first prospective studies to empirically address the intention-behaviour gap in help-seeking for currently untreated mental health problems via the theory of planned behaviour (TPB) (Ajzen 1991, 2002). The results indicate that attitudes and subjective norms towards help-seeking are positively related to help-seeking intentions, which significantly predict subsequent help-seeking behaviour. If participants perceived professional help as something positive and felt that it was recommended by their surroundings (i.e. positive injunctive norms), they were more likely to form help-seeking intentions and actually seek help. Moreover, perceived self-efficacy was also connected to help-seeking intentions and behaviour, thus validating Bandura’s assumptions (2004) about the power of self-efficacy in health promotion. In sum, the TPB is a promising tool to successfully address the intention-behaviour gap in help-seeking for depressive symptoms (Sheeran and Webb 2016).

Overall, the path models explained about 22–23% (pseudo-*R*^2^*)* of variation in help-seeking behaviour which is similar to previous studies on the TPB in health behaviours (Armitage and Conner 2001; McEachan et al. 2011). The main findings are also in line with previous research in the context of help-seeking intentions for depressive symptoms (Bohon et al. 2016; Hui et al. 2015; Mo and Mak 2009; Schomerus et al. 2009a). However, there are also noticeable differences, for example with regard to the impact of subjective norms. In the current study, subjective norms were an important predictor of help-seeking intentions, whereas they did not predict help-seeking intentions in a college sample investigated by Bohon et al. (2016). Yet, in their model, the three main components of the TPB (attitudes, subjective norms and perceived behavioural control) were strongly correlated (*r* = .77 to *r* = .95), which could explain the lack of significance in the full TPB model. Interestingly, their observations are contrasted by two Chinese studies (Mak and Davis 2014; Mo and Mak 2009) that reported a significant direct and indirect effect (via attitudes and perceived behavioural control) of subjective norms on help-seeking intentions. The collectivistic nature and the value of saving face in Chinese culture are considered as explanations for the strong influence of subjective norms. In a similar cross-cultural study, social norms were a strong predictor of choosing informal over formal help, in an Indian sample (Altweck et al. 2015). Thus, social norms are key in mental health prevention, as they seem to influence the readiness and willingness (i.e. intention) of seeking help from a particular source. Consequently, future research needs to test whether promoting social norms favouring professional versus lay help can lead to a cross-cultural increase in help-seeking.

Personal attitudes predicted help-seeking, as well, which is in line with the aspects of the psychological help-seeking process (Rickwood and Thomas 2012; Romano and Netland 2008; White et al. 2018). Evaluating professional help-seeking as being wise and good in the current situation (see Table S1 for the exact wording of the TPB questionnaire) represents readiness to actually seek help. Furthermore, attitudes are also connected to the concept of willingness, as they presume positive outcomes of help-seeking that might even outweigh potential barriers, such as financial costs or the challenge of disclosing personal information in a therapeutic setting. Conceptually, this balance of costs and benefits is also an integral part of other behaviour change models, for instance, the decisional balance of the transtheoretical model of health behaviour change (Prochaska and Velicer 1997) as well as motivational enhancement theory (Miller and Rollnick 2002). However, in lieu of benefits, motivational enhancement theory describes a dissonance between one’s values or goals (e.g. being healthy) and the costs of behaviour change. Hence, an increase in willingness towards behaviour change depends on the congruency between the target behaviour and one’s values (Miller and Rollnick 2002). Consequently, if someone suffers from psychological stress but does not connect help-seeking to (reaching) one’s goals and values (e.g. being healthy), one might refuse external help despite otherwise positive attitudes towards mental health services.

Moreover, the impact of (perceived) barriers to help-seeking on the association between attitudes and intentions and behaviour requires further attention. For example, if treatment is deemed inaccessible, people might express lower intentions of seeking help, despite having positive attitudes towards professional treatment and treatment-seeking. In the TPB, perceived behavioural control inherently reflects such perceptions about barriers. Surprisingly, the findings indicate a small significant effect of self-efficacy on intentions, all other paths of perceived behavioural control were not significant. To comprehend these findings, it is necessary to look at the context of this study and the differentiation of self-efficacy and controllability with regard to help-seeking.

### Providing Context: Mental Health Care in Germany

Since the current study was conducted in Germany, it needs to be discussed in the appropriate cultural context and in the light of previous findings (Schomerus et al. 2009a). The German health care system offers mental health services to most people without extensive waiting periods. In addition, health insurance is mandatory, and public health insurance covers the costs of evidence-based mental health treatment, for instance, cognitive-behavioural therapy (Kamke 1998). Moreover, the German health care system is based on the principle of solidarity meaning that people may access health care services, even if they are not able to financially contribute to their health insurance (e.g. children or unemployed adults). These are obvious differences to other countries, for example the USA, where financial resources play a pivotal role in accessing health care (Clement et al. 2015; Lang 2005; Mo and Mak 2009; Sareen et al. 2007). In addition, referral by a primary care physician is not mandatory for psychiatric or psychotherapeutic treatment; therefore, the Goldberg-Huxley model (Huxley 1996) is only partially applicable.

Regarding the TPB, this might have consequences for perceived behavioural control, as observed: by removing external barriers to help-seeking, such as financial costs, the system seemingly shifts responsibility towards the individual. At the same time, this might reduce variance in self-efficacy, as there are fewer obstacles to overcome so that the decision to seek help does not depend as strongly on the perceived ability on an individual level. Thus, aspects like attitudes and subjective norms appear to be relatively more important for forming intentions (i.e. willingness). Empirically, this observation corroborates the findings of Schomerus et al. (2009a) and is in line with other studies, in which perceived behavioural control was the weakest predictor of help-seeking (Bohon et al. 2016; Mo and Mak 2009) and less important than in physical health behaviours that are connected to additional (physical) barriers (Nudelman and Shiloh 2015). However, this hypothesis requires further research including population level data to examine the interaction between subjective and objective assessments of barriers to help-seeking and individual capability and willingness to seek help.

### The Differentiation of Self-Efficacy and Controllability in Help-Seeking

On a related note, the findings suggest that it is important to differentiate both aspects of perceived behavioural control: Self-efficacy reflects the concept that one is confident to be able to seek professional help (Bandura 2004; Prochaska and DiClemente 1992; White et al. 2018) and controllability means that one feels able to control the execution of a target behaviour, that is help-seeking is entirely up to them. Following this distinction, controllability seems to target the conditions of performing a behaviour, while self-efficacy reflects general capability, potential barriers notwithstanding. However, these aspects are not be confused with locus of control (Ajzen 2002; Kleinberg et al. 2013). For example, if one expresses low controllability of help-seeking, one could attribute it to multiple causes—if attributed internally, for instance, to one’s incompetence, this could lead to less help-seeking but also less self-efficacy (Kleinberg et al. 2013). If attributed externally, for instance, to a lack of treatment options, this does not conceptually affect perceived self-efficacy (Ajzen 2002; Bandura 2004). As a result, both components represent important but different factors within the help-seeking process, which is empirically supported by an observed moderate correlation between them (*r* = .46, *p* < .01). Presumably, self-efficacy transcends controllability in that it contains the subjunctive potential of seeking help, whereas controllability is tied to indicative conditions. Consequently, self-efficacy might be more strongly associated with attitudinal, controllability with perceived structural determinants of help-seeking, such as perceived financial or social barriers which are empirically less impactful—albeit significant—in the help-seeking process than attitudinal factors (Clement et al. 2015; Sareen et al. 2007). Unfortunately, it was not possible to test these assumptions in the current study, because we did not ask participants why they did or did not (intend to) seek professional help for their problems to retrospectively examine perceived facilitators and barriers to help-seeking.

Finally, this distinction has implications for clinical practice. If self-efficacy indeed surpasses controllability in the context of help-seeking, this should be reflected in the communication with patients and in providing preventive education. For example, preventive messages could address the aspects of confidence and voluntariness instead of control and accessibility. Moreover, practitioners could facilitate a differentiation of these aspects by focussing on resource activation in early patient communication. This is particularly important for general practitioners, because they are usually the first point of contact for persons with depressive symptoms (Huxley 1996). Resource activation is a common factor of successful psychotherapy (Gross et al. 2012) and explicitly focuses on individual strengths and potentials instead of barriers. Thus, it might be beneficial to facilitate self-efficacy despite challenging circumstances instead of reducing structural barriers, for instance, by increasing opportunities to access mental health care.

### Limitations

The investigated sample was rather small and not representative; about 54% had previously received psychiatric or psychotherapeutic treatment, wherefore their knowledge and expectancies regarding treatment might differ from the general population. For example, descriptive statistics and path coefficients (see Table S2) suggest that people with treatment experience were more open towards help and more likely to actually seek help for their current problems. In addition, older females were overrepresented in the sample. Taken together, these aspects could indicate an overestimation of help-seeking, as people without treatment experience, males and younger persons are less likely to seek help according to previous research (Gonzalez et al. 2005). In addition, the current study did not assess further precursory aspects of the TPB, such as normative or behavioural beliefs (Ajzen 1991), and therefore could not evaluate their role in the decision to seek help.

Regarding intentions, this study was not able to fully represent the aspect of willingness (Rickwood and Thomas 2012; White et al. 2018), because intention was assessed with a single item per source. Hence, it was impossible to evaluate how precise and concrete intentions were formed. For example, participants with statistically high intentions to seek help might have the general idea to seek help, but they might as well have more elaborate plans, such as talking to a psychotherapist within the next week. The transtheoretical model of health behaviour change (Prochaska and DiClemente 1992; Prochaska and Velicer 1997), for instance, allows for a distinction of these intentions, with the latter (i.e. preparation) being more likely to lead to behavioural implementation than the former (i.e. contemplation). In addition, we did not capture how well intentions actually reflected participants’ personal values and the perceived instrumentality of help-seeking, which is a determining factor of behavioural performance in motivational enhancement theory (Miller and Rollnick 2002). Consequently, future studies should investigate psychological factors across different health behaviour change models to further the understanding of the help-seeking process and to optimise preventive efforts. Finally, behaviour was measured as a dichotomous variable that did not allow for an examination of the empirical referents of help (Cornally and McCarthy 2011), for instance, regarding the type (i.e. instrumental, emotional), amount or duration of help. Mixed methods research could illustrate the qualia of help-seeking and its connections to psychosocial variables as established in the TPB.

#### Methodological Limitations

There are several methodological caveats to this study: Although the TPB questionnaire was based on a study of the German population (Schomerus et al. 2009a), there is no extensive psychometric validation available, for example regarding convergent or discriminant validity of the instrument. The instrument measured self-efficacy with only one item, and controllability with two items; therefore, it was not possible to test psychometric properties of the perceived behavioural control scale. Moreover, although several covariates were considered, this analysis did not include other factors, such as race/ethnicity that may influence help-seeking (Gonzalez et al. 2005). However, this sample was primarily collected in an area, where ethnic differences are less pronounced (about 6.8% of the population have a migration background). Finally, we chose a community sample to investigate determinants of help-seeking in an ecologically valid context in a region with an observed deficit in the utilisation of mental health care. Despite its statistical limitations as opposed to random sampling, for instance, this study may serve as a starting point for larger scale studies with sufficient statistical power to focus on core variables of the help-seeking processes, which were identified and discussed in this study.

## Conclusions and Implications

The theory of planned behaviour (TPB) seems to be a tenable model to explain help-seeking for mental health problems and thus foster help-seeking. Attitudes and subjective norms predict help-seeking intentions, which predict behaviour. Findings are less clear for the perceived ability to seek help, operationalised as perceived behavioural control (a positive association for self-efficacy but a non-significant one for controllability). Future research should apply more extensive measures of the TPB and other health behaviour change models, for example regarding intentions or willingness, to replicate or challenge our observations. Nevertheless, based on these findings, increasing readiness and willingness by strengthening social support of help-seeking and increasing knowledge and attitudes as well as self-efficacy towards treatment in the general population is recommended to foster help-seeking. This is particularly important for general practitioners, who often represent the first contact with the health care system for people with mental health problems.

## Electronic supplementary material

ESM 1 (PDF 165 kb)

ESM 2 (PDF 16 kb)

ESM 3 (PDF 152 kb)
