# Supplementary material for: Ready, Willing and Able? An Investigation of the Theory of Planned Behaviour in Help-Seeking for a Community Sample with Current Untreated Depressive Symptoms
Source: Prev Sci. 2020 Mar 5;21(6):749–60. doi: 10.1007/s11121-020-01099-2 (PMC7366606; doi:10.1007/s11121-020-01099-2)
Supplement: Supplementary file 1 — (PDF 165 kb) [file 11121_2020_1099_MOESM1_ESM.pdf]

Online supplementary material for the following article:

Tomczyk, S.*, Schomerus, G., Stolzenburg, S., Muehlan, H., & Schmidt, S. (revised). Ready, Willing, and Able? An Investigation of the Theory of Planned Behaviour in Help-Seeking for a Community Sample with Current Untreated Depressive Symptoms. *Prevention Science*

Theory of Planned Behaviour Questionnaire

The following questions were adapted from Schomerus et al. (2009a) to measure three core components of the theory of planned behaviour, namely attitudes towards treatment (ATT), subjective norms (SN), and perceived behavioural control (PBC) with its two components perceived self-efficacy (PBC-SE) and perceived controllability (PBC-C). The study was originally conducted in German, therefore we provide the questionnaire in German and in English (translated by the authors).

* Corresponding author: samuel.tomczyk@uni-greifswald.de; University of Greifswald, Institute of Psychology, Department Health and Prevention, Robert-Blum-Str. 13, 17487 Greifswald (Germany); t +49 3834 420 3806, f +49 3834 420 3812

Supplementary Table S1

Theory of Planned Behaviour Questionnaire, Adapted to a Community Sample With Currently Untreated Depressive Symptoms

Item (German)	Item (English)	Scale	*M* (*SD*)
1. Die meisten meiner **Familienmitglieder**, die mir wichtig sind, sind der Meinung, ich sollte mit meinen Beschwerden psychotherapeutische oder medizinische Hilfe suchen.	1. Most **family members** who are important to me think that I should seek medical or psychotherapeutic help for my problems.	SN	3.12 (2.16)
2. Die meisten meiner **Freunde**, die mir wichtig sind, sind der Meinung, ich sollte mit meinen Beschwerden psychotherapeutische oder medizinische Hilfe suchen.	2. Most **friends** who are important to me think that I should seek medical or psychotherapeutic help for my problems.	SN	2.82 (2.04)
3. **Andere Menschen**, die mir wichtig sind, sind der Meinung, ich sollte mit meinen Beschwerden psychotherapeutische oder medizinische Hilfe suchen.	3. **Other people** who are important to me think that I should seek medical or psychotherapeutic help for my problems.	SN	2.60 (2.04)
4. Mit derartigen Beschwerden wird von mir erwartet, professionelle Hilfe zu suchen.	4. With a problem like that, it is expected of me that I seek professional help.	SN	2.93 (2.02)

Mit den geschilderten Beschwerden professionelle Hilfe zu suchen wäre für mich…	Seeking professional help for the problem described would be...	ATT	4.99 (2.00)
5. … sinnlos–sinnvoll	5. …useless–wise		
6. … schädlich–unschädlich	6. …harmful–beneficial	ATT	5.61 (2.00)
7. … gut–schlecht*	7. …good–bad*	ATT	5.50 (1.61)
8. Es liegt nur an mir, ob ich mir mit meinen Beschwerden professionelle Hilfe suche oder nicht.	8. It is entirely up to me whether I seek professional help for my problems.	PBC; PBC-C	6.07 (1.47)
9. Ich bin mir sicher, wenn ich wollte, könnte ich mit meinen Beschwerden professionelle Hilfe in Anspruch nehmen.	9. I am confident that I could seek professional help for my problems if I wanted to.	PBC; PBC-SE	5.47 (1.87)
10. Die Entscheidung, mir für meine Beschwerden professionelle Hilfe zu suchen, liegt ganz allein in meiner Hand.	10. The decision to seek professional help for my problems is entirely up to me.	PBC; PBC-C	6.07 (1.57)
11. Um professionelle Hilfe für meine Beschwerden zu bekommen, brauche ich Unterstützung von außen.[a]	11. I need external support to receive professional help for my problems.[a]	PBC; PBC-C	4.12 (2.37)

Note. [a]Item is reverse coded. Item 11 was excluded from the analysis because of low reliability.
