# Supplementary material for: Ready, Willing and Able? An Investigation of the Theory of Planned Behaviour in Help-Seeking for a Community Sample with Current Untreated Depressive Symptoms
Source: Prev Sci. 2020 Mar 5;21(6):749–60. doi: 10.1007/s11121-020-01099-2 (PMC7366606; doi:10.1007/s11121-020-01099-2)
Supplement: Supplementary file 2 — (PDF 16 kb) [file 11121_2020_1099_MOESM2_ESM.pdf]

Online supplementary material for the following article:

Tomczyk, S.[*], Schomerus, G., Stolzenburg, S., Muehlan, H., & Schmidt, S. (revised). Ready, Willing, and Able? An Investigation of the Theory of Planned Behaviour in Help-Seeking for a Community Sample with Current Untreated Depressive Symptoms. *Prevention Science*

[*] Corresponding author: samuel.tomczyk@uni-greifswald.de; University of Greifswald, Institute of Psychology, Department Health and Prevention, Robert-Blum-Str. 13, 17487 Greifswald (Germany); t +49 3834 420 3806, f +49 3834 420 3812

Supplementary Table S2

Standardized path coefficients of path models of the Theory of Planned Behaviour (TPB) regarding help-seeking from mental health professionals (psychologist, psychotherapist, or psychiatrist) for depressive symptoms in a German community sample (N = 188)

	Model 1 (attitudes, subjective norms, perceived behavioural control)		Model 2 (attitudes, subjective norms, perceived self-efficacy, perceived controllability)	
Variable	Intention	Behaviour	Intention	Behaviour
TPB[a] Attitudes towards treatment	.24***		.23**	
TPB Subjective norms	.26***		.25***	
TPB Perceived behavioural control	.09	.03		
Self-efficacy			.15*	.10
Controllability			-.03	-.05
Age	-.02	.11	-.02	.10
Gender (ref.[b] male)	-.02	.05	-.01	.06
Monthly income	-.04	-.01	-.05	-.02
Secondary education (ref. low)				

Average	-.01	-.16	-.01	-.15
Highest	-.04	-.03	-.03	-.03
Past treatment (ref. no)	.07	.10	.07	.09
Depression severity	.13	.16*	.12	.15

Note. [a]TPB = Theory of Planned Behaviour; [b]ref. = reference category for binary variables;

*p < .05, **p < .01, ***p < .001
